# Indiscriminate Drugging

**Published:** 1856-03

**Authors:** 


### INDISCRIMINATE DRUGGING.

Will the world ever learn, that it is not by the indiscriminate employment of drugs, that disease is removed, or the folly of attempting to decide, for themselves, upon the morbid derangements to which the human body is subject, without any true knowledge of the laws, by which the system is governed in health and disease, and the still greater folly of applying *secret* remedies of whose composition or properties they know nothing, for the restoration of the body to health, in regard to the departures from which, if possible, they know less. How is it possible to understand the human system, little as we know of it, after the labors of thousands of years, without the laborious study of Anatomy, Physiology, and the laws of life? How is it possible to understand the derangements of this wonderful and fearful machinery, without a knowledge of the great science of Pathology—that branch of medicine which investigates the nature of disease—without the time and opportunity, which cannot be possessed by those who do not devote themselves exclusively to the work. But from the obstinacy of the people upon this subject, we sometimes think we are guilty of about as great folly in attempting to change the course of things upon this subject, and nothing but our abiding confidence in the ultimate prevalence of truth upon all subjects, could at all sustain us, in the discouraging task of attempting to diffuse light upon this subject, and though "*faint*, yet pursuing," in the hope that a better day is coming, for those who are to succeed us, if not in our time, we still labor on, and as entirely expressive of our views upon this subject, we adopt some late remarks of the Editor of the Boston Medical and Surgical Journal.

He says, in an article upon "newspaper recommendations of secret remedies," that the state of the matter is: "A great deal of harm is done by advertised patent medicines and nostrums; the immense quantity of them which is swallowed by the public, cannot fail to be detrimental to the health of the community, while any encouragement to their sale tends to retard the progress of the science of medicine, in one of its most important departments—the application ot remedies to the treatment of disease. It is characteristic of the unenlightened to desire a specific for the cure of every disease, and hence one of the difficulties in the practice of medicine, is to *prevent* our patients from taking drugs.

The intelligent physician knows that a *specific* hardly exists in medicine; that the action of remedies is not so much to directly effect a *cure*, as to remove obstacles to that tendency towards restoration, which is inherent in the diseased organization.

While medicine is of great utility in the treatment of disease, no *two* diseases, and almost *no two cases of the same disease*, are ever treated exactly alike, because there will usually be such a variation in the symptoms, as to require some change in the treatment.

Hence it is incorrect to talk of the *cure* of a disease, by any one remedy (with the single exception, perhaps, of Cinchona in Intermittent fever) and absurd to propose to apply one medicine exclusively to two or more distinct diseases."

But in this, as in many other matters connected with the advancement of medical science and a proper understanding by the non-professional of what can, and cannot be accomplished, we have to lay the blame, to a very considerable extent, at the door of those who profess to treat disease, without any real understanding of the *principles*, which are established in medicine, or from an inexcusable carlessness, or indolence in failing to make the proper discrimination, required by varying constitutions, and almost innumerable other circumstances, which modify and change the indications in almost every case of disease; and upon this ground we rarely ever have any standing recipe, regulated by nosological arrangement, however similar may be the usual manifestations, in a large proportion of the group of symptoms, to which any particular name may be attached, and we believe that it is only in this way that we can avoid falling into a plan of routine practice, utterly subversive of every thing like a scientific treatment.

The two great questions at the bedside, which can alone be relied on as suggestive of a judicious treatment of disease, are, what is the pathological condition ? and as deduced from that, what is the therapeutical indication ? withont any reference to a particular case *just like it*, that we may have seen or heard of.

And upon this ground again, we do not attach the importance that some seem to do, to regular works on what is called the "Practice of Medicine"; at the same time not denying, that properly used, they may be of some value to those who are fitted to use them; not, however, as the mere record of any individual experience, except in accordance with scientific truth, being as we believe they are, the fruitful source of empiricism to those who are not prepared to appreciate them by a previous acquisition of the *principles*, which underlie the practical part of our profession.

We are aware that this latter point, opens a wide field and dangerous ground, and that we are liable to be misunderstood in the limited exposition that we can give of our views, in reference to this matter at present, but as a suggestion of our convictions, we present them, and may recur to the subject at some future day.

